# Metabolic Engineering of for Overproduction of Alpha-Ketoglutarate Using Crude Glycerol

**DOI:** 10.1021/acs.jafc.5c04670

**Published:** 2025-07-09

**Authors:** Chung-Jen Chiang, Ju-Hwa Lin, Doan-Thanh Ta, Nguyen Luan Luu, Yi-Cheng Sun, Yun-Peng Chao

**Affiliations:** † Department of Medical Laboratory Science and Biotechnology, 38019 China Medical University, No. 91, Hsueh-Shih Road, Taichung 40402, Taiwan; ‡ Department of Biological Science and Technology, China Medical University, No. 100, Sec. 1, Jingmao Road, Taichung 406040, Taiwan; § Department of Chemical Engineering, 34902 Feng Chia University, 100 Wenhwa Road, Taichung 40724, Taiwan; ∥ Department of Medical Research, China Medical University Hospital, Taichung 40447, Taiwan

**Keywords:** alpha-ketoglutarate, crude glycerol, metabolic engineering, biobased materials

## Abstract

Alpha-ketoglutarate (α-KG) is a multifunctional chemical with significant and wide applications in industry. This study developed a sustainable production of α-KG based on crude glycerol by reprogramming . The metabolic pathways leading to the synthesis of byproducts were blocked, and the anaplerotic route was manipulated to direct the glycolytic flux into the tricarboxylic acid (TCA) cycle. The strain was further engineered by recruiting the α-KG-insensitive *gltA* and removing *mdh* to render the oxidative TCA cycle incomplete. As a result, the engineered strain enabled the overproduction of α-KG. The microbial fermentation was then conducted in a fed-batch mode. By adjusting the glycolytic flux, the producer strain finally produced α-KG with the titer, productivity, and conversion yield on crude glycerol reaching 44 g/L, 1.58 g/L/h, and 0.51 g/g, respectively. Overall, the results indicate that the developed shows promise for producing α-KG from crude glycerol.

## Graphical abstract

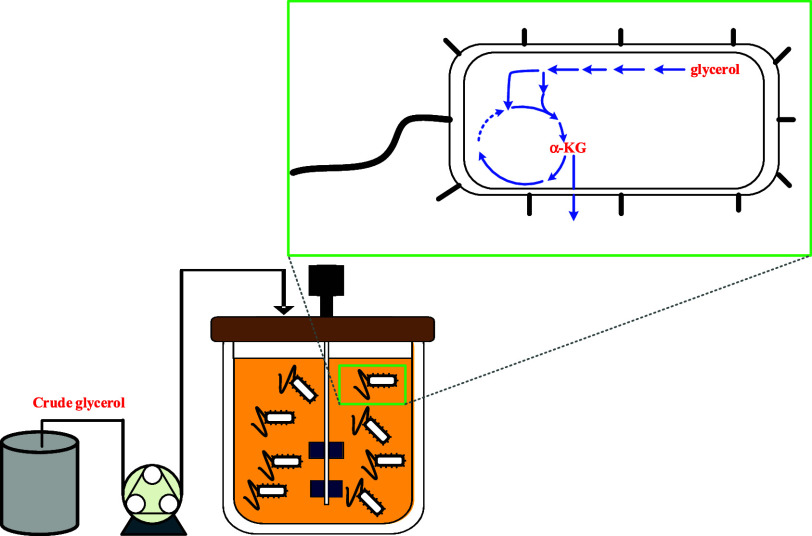

## Introduction

Alpha-ketoglutarate (α-KG) is a multifunctional compound with significant applications in industry. It serves as a building block for the chemical synthesis of N-heterocyclic compounds.^1^ As an animal feed additive, the α-KG supplement improves growth performance, nitrogen utilization, and immunity, as well as reduces intestinal inflammation.^2^ In humans, α-KG exhibits antioxidative activity to improve blood vessel elasticity and functions as a neuroprotective agent in the ischemic pathology of the hippocampus.^3^ It is also involved in the energy metabolism of cells to support mitochondrial function and prevent the incidence of age-related diseases.^4^ The versatile application of α-KG across various industrial sectors has increasingly expanded its market size to meet the evolving needs of consumers.

The production of α-KG is currently undertaken by chemical synthesis using succinic acid and oxalic acid diethyl esters with cyanohydrins.^1^ This production process displays low product selectivity and leads to a production yield of 75%. Moreover, it involves hazardous chemicals and generates toxic waste, which further increases the difficulty of downstream management. To fulfill Sustainable Development Goals (SDGs) with CO_2_ reduction, there is a pressing need to develop an alternative process that is fossil oil-independent, sustainable, and environmentally friendly. In this context, a biotransformation process was designed to synthesize α-KG from glutamate (Glu) by immobilized cells expressing L-amino acid deaminase from .^5^ However, the titer and conversion yield of α-KG were low. The enzymatic synthesis of α-KG from Glu was reported using L-glutamate dehydrogenase from .^6^ This reaction system requires a cofactor recycling system and is hindered by product inhibition. The recombinant L-glutamate oxidase of and catalase were implemented to achieve a high titer of α-KG.^7^ However, the requirement for purified enzymes makes this synthesis route cost-ineffective. Alternatively, the microbial production of α-KG using renewable feedstock provides an appealing solution.

In living cells, α-KG is a precursor metabolite appearing in the tricarboxylic acid (TCA) cycle. The production of glutamate (Glu) from α-KG proceeds through the reaction steps catalyzed by glutamate (Glu) dehydrogenase and Glu synthase, coupled to glutamine (Gln) synthetase. Gln synthetase mediates the reductive amination of Glu to form Gln. Both Glu and Gln further provide the amino group for the biosynthesis of other nitrogen-containing compounds, including amino acids, purines, pyrimidines, and amino sugars, which sustain the metabolic activities of cells.^8^ Moreover, α-KG plays a vital role in coordinating carbon and nitrogen utilization and contributes to maintaining cellular homeostasis.^9^ Studies have been conducted to reveal the fermentative production of α-KG in naturally existing microbes, including , , , , , ssp., and ssp.^10^ In general, these strains’ α-KG titer and productivity are relatively low. This issue was first addressed by the genetic engineering of by inactivating Glu dehydrogenase. However, it resulted in less than 5 g/L α-KG. is an oleaginous yeast, and its auxotrophic mutant for thiamine has been employed and engineered for the overproduction of α-KG.^11^ Thiamine deficiency in inactivates both pyruvate dehydrogenase and α-KG dehydrogenase, leading to the formation of α-KG associated with pyruvate accumulation. The approach by expressing the E1 domain of pyruvate dehydrogenase enabled the increase of α-KG and the reduction of pyruvate.^12^ A similar result was obtained by elevating intracellular acetyl-CoA through the expression of heterologous acetyl-CoA synthase and ATP-citrate lyase.^13^ In addition, several strategies were proposed to trigger the acidogenosis of α-KG. These involve (1) the enhancement of rate-limiting steps in the biosynthesis pathway by coexpression of isocitrate dehydrogenase and pyruvate carboxylase,^14^ (2) the blockage of the competing pathway by mutation of the dihydrolipoamide succinyltransferase domain to attenuate α-KG dehydrogenase activity,^15^ and (3) the increase in α-KG efflux by elevating YlJen5p transporter activity.^16^ These pathway engineering approaches have proven useful in achieving a high titer of α-KG.

 is well-known for its biotechnology-friendly nature. By metabolic engineering, the genetically modified strain has been extensively applied for the mass production of various chemicals.^17^ Crude glycerol is a renewable feedstock that exists in the waste stream from the biodiesel production process. The increasing need for green fuels has led to the excessive production of crude glycerol, with the amount projected to reach around 5 million tons by 2026.^18^ With crude glycerol, we have developed a sustainable production of biobased products in .^19–21^ In this study, the glycerol-based production of α-KG was achieved through pathway engineering of . The resulting strain enabled the production of α-KG with high titer and productivity, indicating the potential application of the production process as developed.

## Materials and Methods

### Plasmid Construction

Plasmid pPhi80-PGlt with the R6K origin is a conditional-replication vector and contains a synthetic operon involving *ppc* of and *gltA* of (Cg*gltA*) under the control of the λP_L_ promoter. This plasmid was constructed using the One-step cloning kit (ZGene Biotech Co., Taiwan). The requirements for primer design followed the manufacturer’s instructions. The *ppc*-borne DNA was first obtained by PCR with primers PPC1 (ggtgatactgagtgtctggggtaatatgaacg) and PPC2 (gcccatatgcattagccggtattacgcatac). The backbone of the pPhi80-Pha plasmid^21^ was amplified by PCR with primers Phi1 (accccagacactgcatatgggctgacctcg) and Phi2 (ataccggctaatcagtatcaccgccagtgg). These two PCR DNAs were then spliced together to give the pPhi-PPC plasmid using the cloning kit. Meanwhile, PCR was applied to amplify the Cg*gltA*-borne DNA with the GLT1-GLT2 primer pair (ctgacctcgagcttccgtaatccggaagag and gtaaaacgacgttagcgctcctcgcgaggaa) and the backbone of the pPhi-PPC plasmid with the Phi3-Phi4 primer pair (aggagcgctaacgtcgttttacaacgtcgtg and gattacggaagctcgaggtcagcccatatg). These two DNAs were then ligated to obtain the pPhi80-PGlt plasmid. With the pPhi80-PGlt plasmid, the DNA containing λP_L_-regulated *ppc* and Cg*gltA* was integrated at the Φ80 *att* site of the cell following the published protocol.^22^

### Strain Development

The strain was cured of D-lactate dehydrogenase from (Lh-*ldh*) as follows. Two homologous DNAs of Lh-*ldh* with a length of around 0.25 kb were amplified by PCR with the primer pairs, including Ldh1-Ldh2 (cttacgctattcgaaaagacg and gaagcagctccagcctacaccttagccttgtccatatc) and Ldh3-Ldh4 (ggtcgacggatcccggaatccgtggtttggactcagg and ctggagaatctggcttttcg). The underlined sequences of primers are complementary to the two terminal regions of the FRT-kanamycin (*kan*)-FRT cassette. The FRT-*kan*-FRT DNA was obtained from the pKD13 plasmid^20^ by PCR with primer KD-1 (gtgtaggctggagctgcttc) and KD-2 (attccggggatccgtcgacc). Three PCR DNAs were then mixed and assembled by overlap extension PCR with the Ldh1 and Ldh4 primers. The PCR reaction produced the passenger DNA consisting of the FRT-*kan*-FRT DNA flanked by two homologous regions of Lh-*ldh*. Following the reported method,^23^ the passenger DNA was electroporated into the host strain expressing λRed. The *kan*-resistant transformants were scored, and the inserted *kan* was then removed by using Flp.

The endogenous *ldhA* was knocked out by P1 transduction according to our protocol.^24^ The P1 lysate was first prepared from the JW1375 (△*ldhA*::FRT-*kan*-FRT) strain by P1*vir*. Following P1 lysate infection, recipient cells were screened for the *kan*-positive phenotype. Colony PCR was performed to verify the transduction event with primers LDH1 (cgtcatcagcagcgtcaacg) and LDH2 (acgctttccagcacaaagc). Flp was subsequently applied to remove the inserted *kan*. Similarly, the elimination of *poxB* and *mdh* genes was conducted by P1 transduction. The donor cells for the preparation of P1 lysate were JW0855 (△*poxB*::FRT-*kan*-FRT) and JW3205 (△*mdh*::FRT-*kan*-FRT) strains. The genotype was verified by colony PCR with primer pairs, including POX1-POX2 (gcaataacgttccggttgtc and accgacaatatactcttcgc) and MDH1-MDH2 (cacggcatgcaaattctgc and gggtattcaggtcaacgatc).

### Production of α-KG

The engineered strains were routinely investigated for α-KG production using Erlenmeyer flasks (125 mL). An optical wavelength of 550 nm (OD_550_) was applied to measure cell density. The seeding culture was prepared with LB medium (5 mL) at 37 °C overnight. After inoculation with seeding cells, the cell culture was incubated in shake flasks containing minimal medium (15 mL) supplemented with 20 g/L crude glycerol (Yeow Hwa Co., Ltd., Taiwan) and 1 g/L yeast extract. The medium composition consisted of salts, including NH_4_Cl (1 g/L), Na_2_HPO_4_ (6.04 g/L), KH_2_PO_4_ (3 g/L), NaCl (0.5 g/L), MgSO_4_·7H_2_O (0.24 g/L), CaCl_2_ (0.022 g/L), and trace minerals, including FeSO_4_·7H_2_O (8 mg/L), Al_2_(SO_4_)_3_·16H_2_O (1.31 mg/L), ZnSO_4_·7H_2_O (0.2 mg/L), CuCl_2_·2H_2_O (0.1 mg/L), NaMoO_4_·2H_2_O (0.2 mg/L), MnCl_2_ (0.74 mg/L), CoCl_2_·6H_2_O (0.07 mg/L), and H_3_BO_4_ (0.05 mg/L).

The bacterial fermentation was conducted using a BioFlo-120 fermenter (2 L) equipped with a BioFlo-120 Bioprocess Control Station (Eppendorf, Germany). The seeding culture was prepared with cells in shake flasks containing minimal medium and crude glycerol (20 g/L). After inoculation into the fermenter, cell fermentation was carried out with minimal medium plus crude glycerol as indicated. The fermentation conditions were maintained at 37 °C, and a solution containing KOH (178.3 g/L) plus MgCO_3_ (126.5 g/L) was used to control the pH at 7. The DO was controlled automatically using the cascading agitation mode, and oxygen was supplied by purging air at 1 vvm.

### Analytical Methods

The culture broth was collected for analysis after the bacteria were harvested by centrifugation. High-performance liquid chromatography (HPLC) equipped with a UV detector at 210 nm was used for the measurement of organic acids. The liquid samples were injected into the Aminex HPX-87H ion exclusion column (Bio-Rad, USA), and the mobile phase (0.0085 N H_2_SO_4_) was pumped at a flow rate of 0.3 mL/min. Glycerol was determined by HPLC equipped with an ICSep ICE-ION-300 column (Transgenomic, USA) and a refractive index detector. The mobile phase (0.0085 N H_2_SO_4_) at 0.6 mL/min was applied for the analysis.

## Results and Discussion

### Development of the Producer Strain

The EcoB-140 strain was previously developed for the fermentative production of D-lactate using crude glycerol.^20^ This producer strain harbored a genomic copy of the heterologous Lh-*ldh* and was deprived of *mgsA* and *dld* to ensure the enantiomeric purity of D-lactate. Its glycerol metabolism was further improved by the enhanced expression of *gldA* and *dhaKLM* involved in the fermentative dissimilation of glycerol. To reduce carbon waste, the strain was modified by eliminating *adhE*, *frdA*, *pflB*, and *pta*, which are responsible for synthesizing ethanol, formate, succinate, and acetate (Figure 1). Our previous study showed that the production of α-KG was detected in the strain under the condition of high glycerol levels and available oxygen.

**Figure 1:**
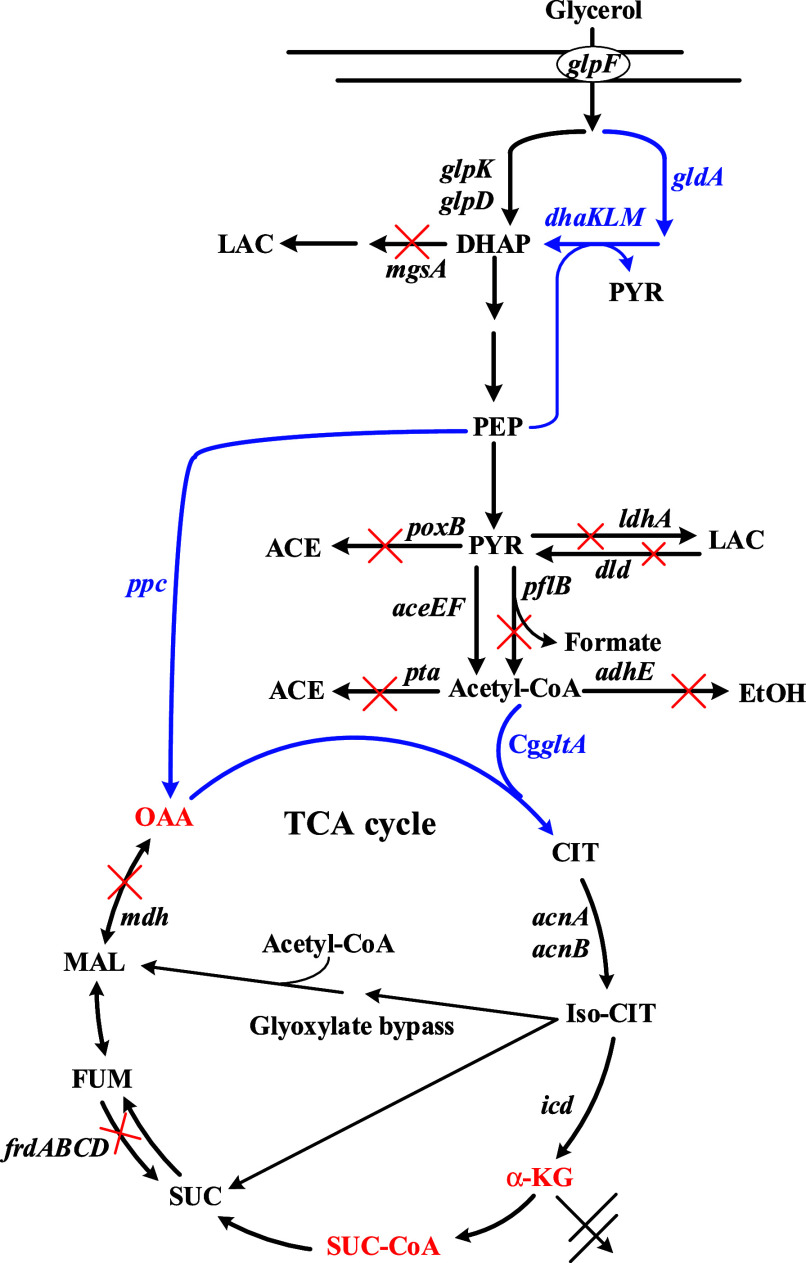
Central metabolic pathways leading to the synthesis of α-KG in . The enhanced pathways are highlighted in blue. The blocked pathways are crossed (X) as indicated. Precursor metabolites in the TCA cycle are shown in red. Genes of interest include: *aceEF*, pyruvate dehydrogenase; *acnA*, *acnB*; aconitase, *adhE*, aldehyde-alcohol dehydrogenase; Cg*gltA*, *citrate synthase* of ; *dld, FAD-linked* D-lactate dehydrogenase*; dhaKLM,* dihydroxyacetone kinase operon*; frdABCD*, fumarate reductase operon*; gldA*, glycerol dehydrogenase; *glpD*, glycerol 3-phosphate dehydrogenase; *glpK*, glycerol kinase; *icd*, isocitrate dehydrogenase; *ldhA*, NAD-linked D-lactate dehydrogenase; *mgsA*, methylglyoxal synthase; *poxB*, pyruvate oxidase; *ppc*, PEP carboxylase; *pflB*, pyruvate-formate lyase; *pta*, phosphate acetyltransferase; Abbreviations of metabolites: ACE, acetate; α-KG, α-ketoglutarate; CIT, citrate; DHAP, dihydroxyacetone phosphate; EtOH, ethanol; FUM, fumarate; LAC, lactate; MAL, malate; OAA, oxaloacetate; PEP, phosphoenolpyruvate; Iso-CIT, isocitrate; PYR, pyruvate; SUC, succinate. SUC-CoA, succinyl-CoA.

This study was initiated with the EcoB-140 strain. To improve α-KG production, the strain was engineered by inactivating Lh-*ldh* and endogenous *ldhA* to curtail carbon waste. The engineered strain (EcKG-1) was cultured in a conical flask with vigorous shaking. As shown in Figure 2, this strain enabled the production of α-KG associated with acetate. In contrast, D-lactate was a major product of the parental strain (EcoB-140). The results indicate that blockage of the synthetic pathway leading to D-lactate increases the intracellular level of intermediate metabolites in glycolysis. As revealed in Figure 1, PEP carboxylase (encoded by *ppc*) mediates the carboxylation of available phosphoenolpyruvate (PEP), replenishing oxaloacetate (OAA) in the tricarboxylic acid (TCA) cycle. Catalyzed by citrate synthase (encoded by *gltA*), the condensation of OAA with acetyl-CoA produces citrate, which is further oxidized to synthesize α-KG. Meanwhile, pyruvate oxidase (encoded by *poxB*) functions to decarboxylate available pyruvate to form acetate in the *pta*-deficient strain.

**Figure 2:**
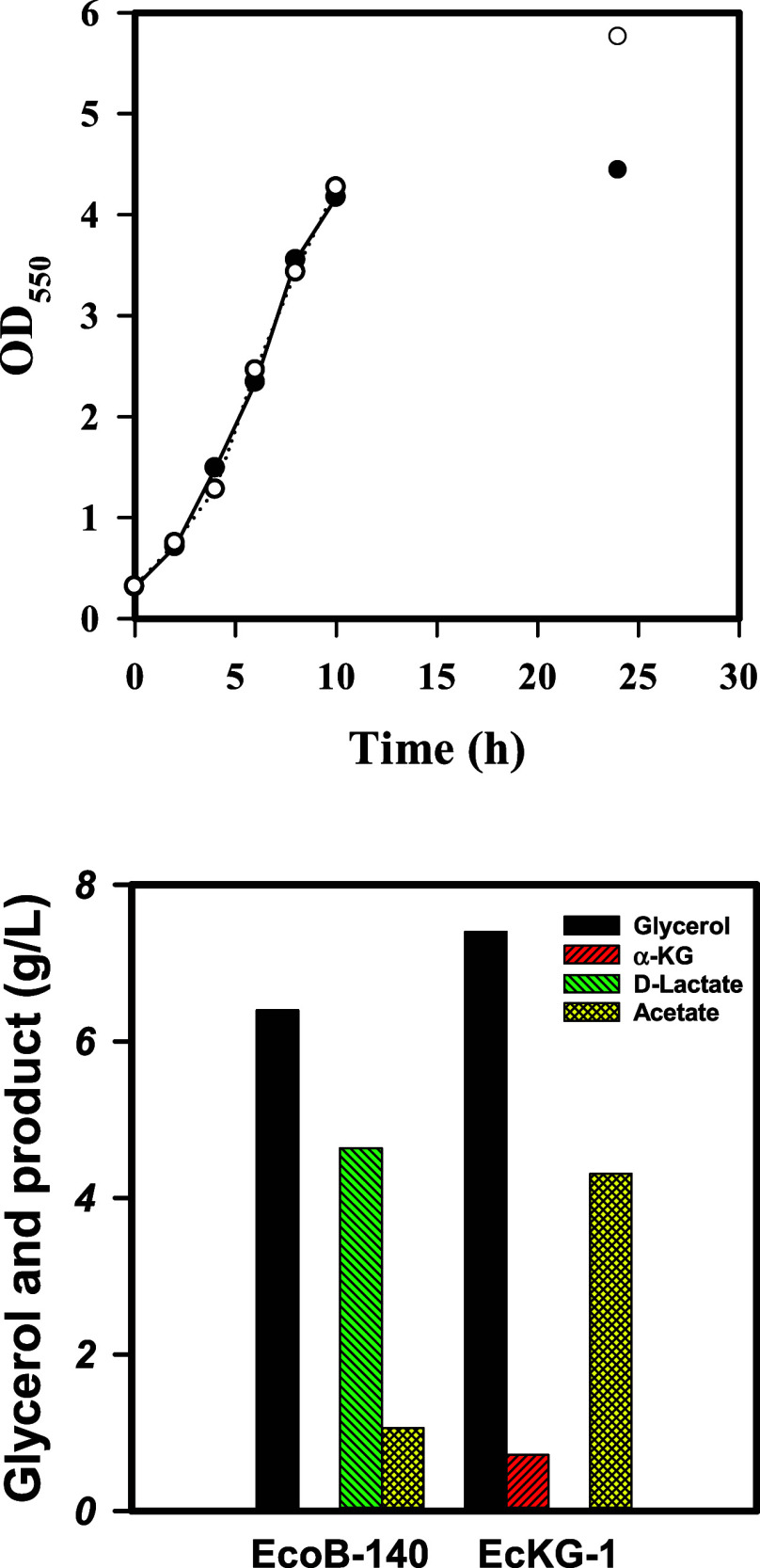
The α-KG production in engineered . strains were cultured in shake flasks containing crude glycerol (10 g/L) for 24 h. The cell growth (OD_550_) was monitored along the time course, and the culture broth was analyzed at the end of the experiment (bottom). The experiment was duplicated. The typical growth profiles were shown for EcKG-1(○) and EcoB-140 strain (●) (top). The HPLC analysis showed the consumption of crude glycerol and the production of α-KG, D-lactate, and acetate (bottom).

### Improvement of the α-KG Producer

The expression of *poxB* is induced in glycerol-grown upon entering the stationary growth phase.^25^ In addition, acetate is reutilized to form acetyl-CoA through the pathway involving acetyl-CoA synthetase (encoded by *acs*). The occurrence of acetate overflow in the EcKG-1 strain likely suggests that the activity of the PoxB-mediated pathway outweighs that of the Acs-involved pathway. Accordingly, *poxB* of the EcKG-1 strain was eliminated to give the EcKG-2 strain. The shake-flask culture was carried out in a similar way. The results showed that the EcKG-2 strain mainly produced pyruvate, and the production level of α-KG remained roughly unaffected (Figure 3)

**Figure 3:**
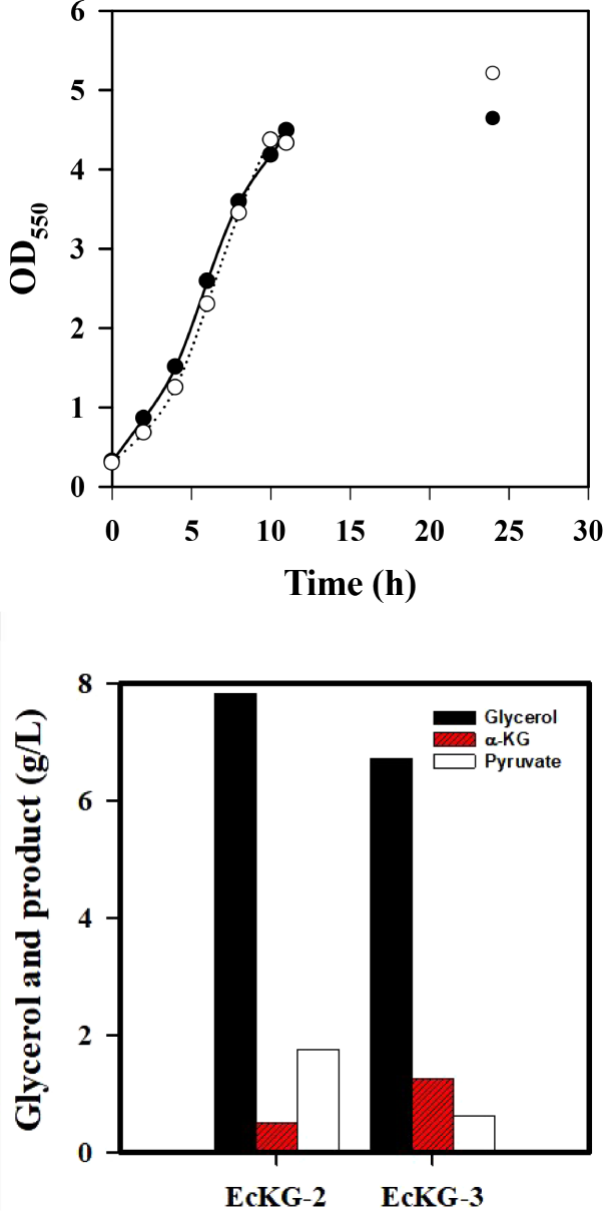
Improved production of α-KG with the genetically modified . strains were cultured in shake flasks containing crude glycerol (10 g/L) for 24 h. The cell growth (OD_550_) was followed along the time course, and the culture broth was analyzed at the end of the experiment. The experiment was duplicated. The typical growth profiles were shown for EcKG-2(●) and EcKG-3 strain (○) (top). The HPLC analysis showed the consumption of crude glycerol and the production of α-KG and pyruvate (bottom).

It is appealing to recycle pyruvate for the improved production of α-KG. OAA and acetyl-CoA serve as precursors for synthesizing α-KG in the TCA cycle. In , Ppc plays a significant role in the anaplerotic route that supplies OAA. The available acetyl-CoA and aspartate render the regulation of Ppc activity ultrasensitive to the fructose-1,6-bisphosphate (FBP) level.^26^ GltA catalyzes the first reaction step in the TCA cycle, responsible for the formation of citrate from OAA and acetyl-CoA. The regulation of GltA activity is subject to the allosteric inhibition by NADH^27^ and the competitive binding of α-KG at the active site.^28^ In contrast to GltA, GltA from (encoded by Cg*gltA*) displays insensitivity to NADH and α-KG.^29^ Therefore, the EcKG-2 strain was engineered by enhancing the expression of *ppc* and recruitment of Cg*gltA*. This was carried out by genomic insertion of the DNA containing λP_L_-regulated *ppc* and Cg*gltA*. The resulting strain (EcKG-3) was shown to double the production yield of α-KG and largely reduce the pyruvate level (Figure 3).

### Large-Scale Production of α-KG

The synthesis of α-KG originates from the TCA cycle. The activity of the TCA cycle is coupled to oxygen availability.^8^ To control the dissolved oxygen (DO) level, the EcKG-3 strain was cultured in a bench-scale fermenter. The cell culturing was conducted in batch mode under aerobic conditions. As shown in Figure 4A, the strain consumed all crude glycerol after the aerobic culturing proceeded for 28 h. The production titer of α-KG reached 16.7 g/L, and pyruvate (2.6 g/L) was detected in the culture broth at the end of the experiment.

**Figure 4:**
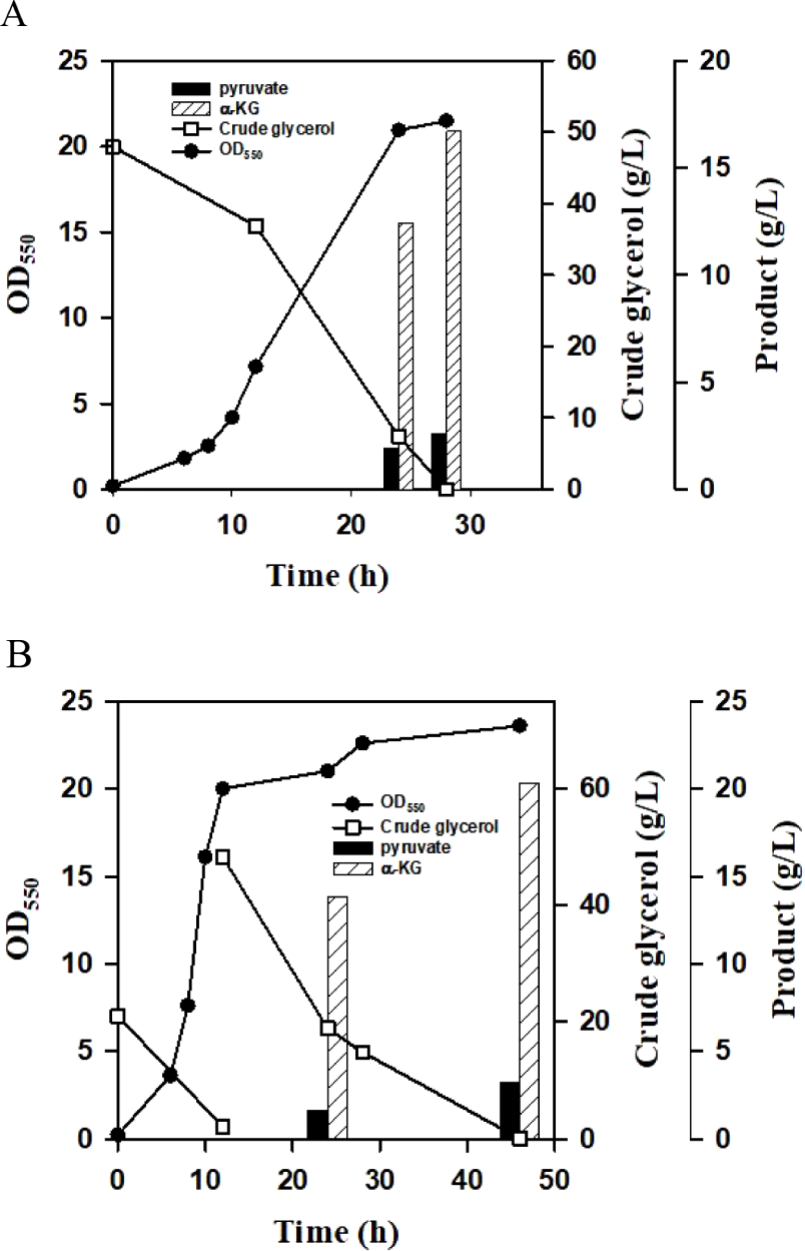
The α-KG production using a bench fermenter. (A) The batch fermentation was conducted with crude glycerol (50 g/L) under the aerobic condition. The fermentation proceeded for 28 h, and the culture broth was sampled and analyzed by HPLC. The experiment was duplicated. The typical fermentation profiles were shown for EcKG-3 strain. (B) The two-stage fermentation was carried out as indicated. At first, the aerobic culturing was conducted with 20 g/L crude glycerol. The supplement of crude glycerol (50 g/L) was then administered, and the culture condition was shifted to the DO-limited level. The experiment was duplicated. The typical fermentation profiles were shown for EcKG-3 strain.

In fermentative , the TCA pathway is incomplete and operates in the reductive direction. It involves one route for the oxidation of citrate to form α-KG and another for the reduction of OAA to synthesize succinyl-CoA.^30^ In the reductive route, fumarate reductase (encoded by *frdA*) mediates the conversion of fumarate to succinate. The EcKG-3 strain indeed lacks *frdA*. This, in turn, conserves OAA, which favors the oxidation of citrate to form α-KG. Therefore, a two-stage fermentation was conducted with the EcKG-3 strain. The strain was first grown on crude glycerol under aerobic conditions. As shown in Figure 4B, crude glycerol was completely consumed at 12 h. Crude glycerol (50 g/L) was then added to the culture broth, followed by controlling the DO level to 3% of the saturated level. The entire fermentation proceeded for 46 h, and the strain produced α-KG with a titer of 20 g/L.

### Fed-Batch Fermentation of α-KG

As illustrated above, the reductive TCA pathway favors the production of α-KG. However, it reduces α-KG productivity. This issue was addressed by inactivating *mdh* encoding malate dehydrogenase, to render the oxidative TCA pathway incomplete (Figure 1). Mdh catalyzes the reversible oxidation of malate to form OAA. The oxidative TCA pathway lacking *mdh* still functions to synthesize precursor metabolites for biosynthetic needs but cannot supply OAA. Through the Ppc-involved pathway, the carbon flux that enters the TCA pathway could be utilized to synthesize α-KG. The EcKG-4 strain was thus obtained by eliminating *mdh* in the EcKG-3 strain. The batch culturing of the EcKG-4 strain was carried out under aerobic conditions. As shown in Figure 5A, the strain produced around 24 g/L of α-KG with 3 g/L of pyruvate at the expense of all crude glycerol.

**Figure 5:**
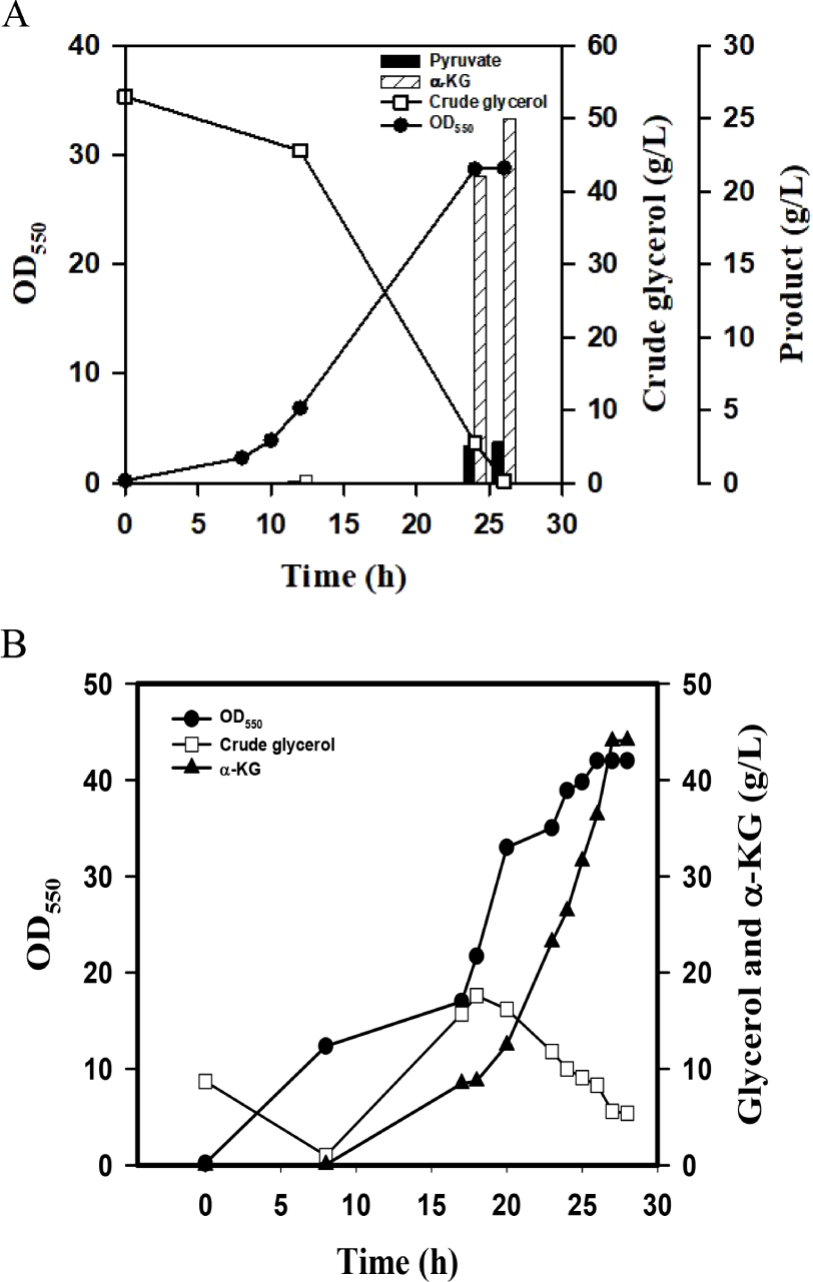
The α-KG production by fed-batch fermentation. (A) The production performance of EcKG-4 strain was first investigated with the batch fermentation. This was conducted with crude glycerol (50 g/L) under the aerobic condition. The fermentation proceeded for 26 h, and the culture broth was sampled and was analyzed by HPLC. The experiment was duplicated. The typical fermentation profiles were shown for the strain. (B) The fed-batch fermentation was carried out as follows. First, the aerobic culturing was conducted with 10 g/L crude glycerol. Then, crude glycerol at a constant rate was continuously fed into the culture broth. The DO level was maintained at 30% of the saturated level throughout the experiment. The culture broth was sampled for the analysis along the time course. The experiment was duplicated. The typical fermentation profiles were shown for the EcKG-4 strain.

The fed-batch fermentation provides a useful way to increase cell density and the production of fermentation products. As illustrated above, there was still a detectable level of pyruvate. The strategy of a constant feed rate was adopted to adjust the glycolytic flux in the cell. Therefore, the aerobic culturing of the EcKG-4 strain was conducted in a fed-batch mode. First, crude glycerol of 10 g/L was used for cell growth (Figure 5B). After culturing for 8 h, crude glycerol at a rate of 5 g/(L·h) was continuously pumped into the culture broth. The production of α-KG started to accumulate over time. Finally, the strain enabled the production of 44 g/L α-KG.

 has been explored for the cost-effective production of α-KG using glycerol (Table 1). In one study, the fermentation approach was conducted with a two-stage pH control and the constant feeding of pure glycerol. The thiamine-auxotrophic WSH-Z06 was shown to produce α-KG with a titer of 66.2 g/L and productivity of 0.35 g/L/h.^31^ Meanwhile, there was an associated production of 26.1 g/L pyruvate. The presence of pyruvate causes difficulty in the downstream purification of α-KG. This issue was further addressed by exploiting pyruvate carboxylase (PCL) to channel the pyruvate flux into the TCA cycle. The pyruvate level was reduced to 13.5 g/L for RoPYC2, which carried the PCL gene (RoPYC2) from , and α-KG production reached 62.5 g/L.^32^ In addition to PCL, the fumarase gene was introduced into H355. The physiological role of fumarase mediates the conversion of fumarate to malate in the TCA cycle. The recombinant strain enabled the production of 138 g/L α-KG from raw glycerol (glycerol content 96%), and the accompanying production of byproducts was lowered to 5.9% of total organic acids.^33^ Isocitrate dehydrogenase (IDH) is responsible for the formation of α-KG from isocitrate. Equipped with PCL and IDH, the modified H355 produced 186 g/L α-KG and 8 g/L pyruvate.^14^ The productivity and conversion yield of α-KG were 1.59 g/L/h and 0.36 g/g, respectively. Nevertheless, the industrial production of α-KG using remains challenging due to the complicated operation of thiamine-limited fermentation, significant accumulation of byproducts, and long fermentation time.^11^

 was previously employed for the overproduction of α-KG. To reduce the production of byproducts, was deprived of undesired genes encoding lactate dehydrogenase (*ldhA*), pyruvate formate lyase (*pflB*), pyruvate oxidase (*poxB*), phosphotransacetylase (*pta*), acetate kinase (*ackA*), fumarate reductase (*frdBC*), and fumarase (*fumABC*). The genes involved in the metabolic pathways favoring the synthesis of α-KG were overexpressed to redistribute the carbon flux. These included the endogenous *acs*, *gltA*, *acnA* (encoding aconitase), *icd* (encoding isocitrate dehydrogenase), and the foreign PCL gene. Consequently, the α-KG production and productivity for glucose-grown were 32.2 g/L and 0.54 g/L/h, respectively.^34^ In this study, was reprogrammed for the desired trait in several steps. The unnecessary genes were deleted to curtail carbon waste, mainly including *adhE*, *frdA* (encoding fumarate reductase), *ldhA*, *mdh*, *mgsA* (encoding methylglyoxal synthase), *pflB*, *poxB*, and *pta*. The glycerol metabolism of the cell was improved by enhancing the expression of *gldA* and *dhaKLM*. Moreover, the glycolytic flux was directed into the TCA cycle by overexpression of endogenous *ppc* and heterologous *gltA*. With crude glycerol (glycerol content 63.5%), the recombinant finally enabled the production of 44 g/L α-KG without pyruvate and acetate (Figure 5B). This resulted in a productivity of 1.58 g/L/h and a conversion yield of 0.51 g/g.

**Table 1:** Summary of the α-KG Production in Various Microorganisms

Strains	Substrate	Titer (g/L)	Productivity (g/L/h)	Conversion Yield (g/g)	References
WSH-Z06	Pure glycerol	66.2	0.35	0.66	^31^
RoPYC2	Pure glycerol	62.5	0.43	0.63	^32^
H355	Raw^a^ glycerol	138	1.44	0.51	^33^
T5	Raw^a^ glycerol	186	1.59	0.36	^14^
△4-PCAI	Glucose	32.2	0.54	0.32	^34^
EcKG-4	Crude^a^ glycerol	44	1.58	0.51	This study

a The glycerol content in raw glycerol and crude glycerol is 96% and 63.5%, respectively.

In summary, our engineered strain exhibits superior production selectivity for α-KG (in terms of the conversion yield). Its α-KG productivity is comparable to that of (Table 1). Notice that the crude glycerol used herein has a low glycerol content. The effect of the impurities in crude glycerol on α-KG production remains unclear. Nevertheless, the α-KG producer usually produced a detectable level of pyruvate (Figures 4 and 5A). This implies that the rate-limiting steps for α-KG synthesis likely appear in the developed strain. In the TCA cycle, AcnB serves as the primary catabolic enzyme responsible for converting citrate to isocitrate. Its prosthetic group contains iron and is susceptible to oxygen.^35^ Isocitrate is at the junction of the TCA cycle and the glyoxylate bypass pathway. The metabolite levels affect the phosphorylation state of isocitrate dehydrogenase, which determines the flux distribution at the junction node.^33^ In addition, α-KG plays a regulatory role in maintaining cellular homeostasis. It inhibits PtsI, GltA, and GlnB ,^9,28,36,37^ which are involved in the sugar transport system, the TCA cycle, and the Ntr regulon in response to nitrogen availability. Future work will require deciphering the underlying mechanism and identifying unknown control targets. Overall, the current results indicate that the newly developed strain is promising for α-KG production using crude glycerol.
